# Prevalence, Awareness, Treatment, and Control of Hypertension in Young Adults (20–39 Years) in Kerala, South India

**DOI:** 10.3389/fcvm.2022.765442

**Published:** 2022-04-18

**Authors:** Zachariah Geevar, Mangalath Narayanan Krishnan, Krishnannair Venugopal, Ganesh Sanjay, S. Harikrishnan, Padinhare Purayil Mohanan, G. K. Mini, Kavumpurathu Raman Thankappan

**Affiliations:** ^1^Mother Hospital, Thrissur, India; ^2^Ahalia Hospital, Abu Dhabi, United Arab Emirates; ^3^Department of Cardiology, Pushpagiri Medical College, Tiruvalla, India; ^4^Department of Cardiology, Sree Chitra Tirunal Institute for Medical Sciences and Technology, Thiruvananthapuram, India; ^5^Westfort Hitech Hospital, Thrissur, India; ^6^Department of Public Health, Global Institute of Public Health, Thiruvananthapuram, India; ^7^Department of Public Health, Central University of Kerala, Kasaragod, India

**Keywords:** hypertension in young adults, hypertension in India, treatment and control of hypertension, prevalence of hypertension, prevalence of hypertension Kerala, high blood pressure India

## Abstract

**Objective:**

We sought to study the prevalence of hypertension and the levels of awareness, treatment and control of hypertension in the young adults in Kerala, India compared to older adults.

**Methods:**

We identified 1,221 young adults (men 36.7%) in the age group 20–39 years from the 5,150 participants of the Cardiological Society of India Kerala Coronary artery disease (CAD) and its Risk factors Prevalence (CSI Kerala CRP) Study. We determined prevalence and levels of awareness, treatment and control of hypertension among them compared to older adults

**Results:**

We found that among the young adults, 11.2% had hypertension and 33.3% had prehypertension. Hypertension was nearly three times more prevalent among men than women (20.5 vs. 7.5% *p* < 0.001) while in older adults there was no difference between men and women in its prevalence. Male sex (OR 3.36, 95% CI 2.15–5.25 *p*-value <0.001), urban residence (OR 2.21, 95% CI 1.52–3.22 *p*-value <0.001), abdominal obesity (OR 1.74, 95% CI 1.06–2.87 *p*-value 0.028) and hypercholesterolemia (OR 1.64 95% CI 1.12–2.40 *p-*value 0.011) were significant factors favoring hypertension in the young adults. Awareness and treatment of hypertension were significantly poor among younger adults compared to older adults. In young adults, awareness, treatment and control of hypertension were significantly lower among men compared to women (23.9 vs. 51.7% *p*-value 0.001, 12.0 vs. 25.9% *p*-value 0.045, and 18.5 vs. 37.9% *p*-value 0.012, respectively). Participants who had checked blood pressure at least once during the previous year had significantly better awareness and treatment (58.7 vs. 24.0% and 41.3 vs. 19.2%, respectively).

**Conclusions:**

We found that one eighth of young subjects had hypertension with three times higher prevalence of hypertension among men compared to women. Awareness, treatment and control of hypertension were less among young adults and worse in young men compared to young women. Identifying hypertension and measures to control it are important and should be specifically targeted to young men.

## Introduction

Hypertension ranks as the second leading risk factor for men and the leading risk factor for women globally, accounting for about 90 million disability adjusted life years (DALYs) among women and about 125 million DALYs among men (1). Ischemic heart disease (IHD) is the largest source of DALYs attributable to high systolic blood pressure (SBP), followed by haemorrhagic stroke and ischemic stroke (1).

The overall prevalence of hypertension among adults in India is about 30% with urban prevalence of 34% and rural prevalence of 28% (2). Unfortunately, only 25% of rural and 38% of urban Indians with hypertension are being treated. Control rate of hypertension in India is dismal and only one-tenth of rural and one-fifth of urban Indian hypertensive population have their blood pressure (BP) under control (2).

India is a young country and as per census 2011, around 32% of its 1.2 billion people are young adults (age group 20–39 years) (3). Assuming a prevalence of hypertension of around 7% among young adults (20–39 years age group) (4), this translates to around 27 million young adults with hypertension in India. It is known that elevated blood pressure during young adulthood causes vascular damage that result in clinical events and mortality later in life (5).

It is found that awareness, treatment and control of high blood pressure among young adults with hypertension are very low (6). There is also scant information about the most effective treatment strategy for younger patients with hypertension because most clinical trials only include patients over 50 years of age (7).

It is important to study the prevalence and levels of awareness, treatment and control of hypertension among young adults. There have been only a few isolated epidemiological studies reporting prevalence and levels of awareness, treatment and control of hypertension in selected populations in India. However, no studies have been carried out looking at these parameters in young adults in India. Kerala, the southernmost state in India is the most advanced among Indian states with regard to human development index and it is widely believed that what happens in Kerala could be an indicator of what is going to happen in other states in India. We have already reported that prevalence of definite coronary artery disease in Kerala is 3.5% and prevalence of major risk factors for coronary artery disease (CAD) like diabetes, hypertension, smoking and hypercholesterolemia is high (8).

Here we report the prevalence of hypertension among young adults who were participants of the Cardiological Society of India Kerala Coronary Artery Disease and its Risk factors Prevalence Study (CSI Kerala CRP Study). We are also presenting the levels of awareness, treatment and control of hypertension among the younger hypertensive participants in this population when compared to older adults with hypertension.

## Methods

A detailed description of the design, sample, and methods of CSI Kerala CRP study has already been published (9). Briefly, this was a cross-sectional community-based survey during the period from January to June 2011. The main objective was to determine the prevalence of CAD and its risk factors in rural and urban areas of the state of Kerala, India, among men and women between the age 20 and 79 years. We chose 3 districts in the state to represent north, central and south regions of Kerala. For the urban sample, we randomly selected one municipal ward within the municipal corporation of the selected district and then utilizing the 2010 voters list randomly selected a geographical area within each municipal ward to capture ~500 households. For rural sample we randomly selected two gramapanchayats for each district and one panchayat ward was randomly selected within each of these. All the households in the selected panchayat wards were surveyed. Within the selected household, if there was only one person in the age group 20–59 years, that person was selected. If there were more than one person in that age group, we used the Kish method to select one individual for the study. All individuals aged 60–79 years in the household were included in the study.

A sub-sample of young men and women between the age 20 and 39 years constituted participants for the present study.

We collected data using a pre-tested interview schedule. Information on basic socio-economic and demographic details, smoking, physical activity, dietary habits, and history of hypertension, dyslipidaemia, diabetes mellitus and history of CAD were recorded. Blood pressure was measured with electronic BP apparatus (model 1A2, Omron Corporation, Shimogyo-ku, Kyoto, Japan), on the left arm resting on a table at heart level in sitting position after the subject having rested for at least 15 min. We made sure that the participants did not drink coffee or tea, or smoked within half hour of BP measurement. Three readings were taken 3 min apart and the mean of the last two readings was recorded as the final BP (10). Height was measured by wall-mounted stadiometer (Model 206, Seca, Hamburg Germany) to the nearest centimeter. Weight was measured with portable electronic weighing scale (Model HN 283, Omron Corporation, Shimogyo-ku, Kyoto, Japan). Waist circumference was measured at the midpoint between the iliac crest and the lower margin of the ribs using a non-stretchable measuring tape with participants standing erect in a relaxed position with both feet together. Blood samples for plasma glucose and lipid panel investigations were obtained from participants after 10–12 h of fasting.

### Definitions

We defined hypertension as blood pressure ≥140 mm of Hg systolic and/or ≥90 mm of Hg diastolic and/or as current use of antihypertensive medication (11). Prehypertension was diagnosed if the measured SBP was between 120 and 139 mm Hg and or if diastolic BP was between 80 and 89 mm Hg provided the participants were not on anti-hypertensive medications. We defined diabetes as fasting blood glucose value of ≥7 mmol/L and/or if there was current use of medications for diabetes (12). Hypercholesterolemia was defined as a total cholesterol of ≥5.2 mmol/L or as current use of lipid lowering medication (13). We categorized body mass index (BMI) as normal (18.0–22.9 kg/m^2^), overweight (23.0–24.9 kg/m^2^), or obesity (≥25 kg/m^2^); abdominal obesity was defined as a waist circumference of ≥90 cm in men or ≥80 cm in women (14). Physical activity level was classified into sedentary and non-sedentary. All participants whose jobs involved physical activity or those performing activity involving physical effort for at least 30 min a day for a minimum of 5 days a week (household activities involving physical effort, walking to and from work, or leisure-time physical activities) were considered non-sedentary. All others were classified as sedentary. We defined current tobacco users as those who have used tobacco in the previous 1 month. We defined current alcohol users as those who have consumed alcohol containing beverages at least once in the past 1 year.

We assessed dietary habits through survey questions about overall dietary status (vegetarian vs. non-vegetarian), fruit and vegetable intake, and intake of salty foods specific to Kerala. We classified participants as having high salt intake if they answered yes to two of the three questions related to salt intake (adding salt to rice during cooking or at the table, consuming salty dry fish servings at least once per week or consuming high salt add-on items to rice at least five times per week). We defined below poverty line as those possessing a ration card from the public distribution system of the state indicating Below Poverty Line (BPL) status based on criteria set by the government. We assessed knowledge level about risk factors for CAD by seven questions related to hypertension, diabetes, smoking and dietary habits and classified the participants as good, satisfactory or poor knowledge level of risk factors (correctly answered 5–7 questions, 2–4 questions, 0–1 questions, respectively) (Appendix 1). We also collected information whether participants have checked their blood pressure at least once during the past 1 year.

### Statistical Analysis

The baseline characteristics were summarized using frequency and percentage. Awareness rate was calculated as the proportion of persons with SBP ≥ 140 mmHg or DBP ≥ 90 mmHg with a prior diagnosis by a physician or the use of any antihypertensive medicine as reported by the respondent. The treatment rate was calculated as the proportion of persons who had used anti-hypertensive medication, among the hypertensives. Control rate of hypertension was the proportion of hypertensives who had SBP <140 and DBP <90 mmHg. Chi-square tests were used to determine the association between categorical variables. Based on multivariate analysis, we used a multivariate logistic regression model to determine the factors associated with the prevalence of hypertension in young adults. Results are presented as odds ratios (ORs) with 95% confidence intervals (CIs). Age-adjusted hypertension prevalence, treatment and control were calculated based on the standard World Health Organization (WHO) world population. The minimum statistical significance level was fixed as *p* < 0.05. The data were analyzed using SPSS version 21.0 (IBM SPSS Statistics for Windows, Version 21.0. Armonk, NY: IBM Corp).

## Results

Of the 5,167 men and women who participated in the CSI Kerala CRP Study, 17 participants were excluded due to incomplete biochemical values and anthropometric measurements. Of the remaining 5,150 participants, 1,221 were young adults in the age group 20–39 years and were available for analysis. There were missing values for obesity in six, abdominal obesity in eleven, diet habits in three, diabetes in 37 and cholesterol levels in 45 participants. The baseline characteristics of the young adults, older adults and the overall study population are given in Table 1. We found that the proportion of women were higher among the younger participants of the study, compared to older adults. In general there were more participants from rural areas than from urban areas and this difference was prominent among the younger participants. Younger adults were better educated than their older counter parts.

**Table 1:** Baseline characteristics of young adults (20–39 years), older adults (40–79 years), and overall adults (20–79 years).

**Characteristics**	**Young adults** **(20–39 years)** ***N* = 1221 *n* (%)**	**Older Adults** **(40–79 years)** ***N* = 3,929 *n* (%)**	**Overall** **(20–79 years)** ***N* = 5,150 *n* (%)**	***P-*value**
**Socio-economic and demographic**				
**Sex**				
Women	773 (63.3)	2,316 (58.9)	3,089 (60.0)	0.007
Men	448 (36.7)	1,613 (41.1)	2,061 (40.0)	
**Place of residence**				
Rural	767 (62.8)	2,142 (54.5)	2,909 (56.5)	<0.001
Urban	454 (37.2)	1,787 (45.5)	2,241 (43.5)	
**Education**				
Up to school education	709 (58.1)	3,233 (82.3)	3,942 (76.5)	
Above school education	512 (41.9)	696 (17.7)	1,208 (23.5)	<0.001
Below poverty line	503 (41.2)	1,472 (37.2)	1,975 (38.3)	0.020
**Risk factors**				
Obesity	497 (40.8)	1,575 (40.1)	2,072 (40.3)	0.688
Abdominal obesity	712 (58.5)	2,525 (64.4)	3,237 (63.0)	<0.001
Sedentary physical activity	183 (15.0)	1,076 (27.4)	1,259 (24.4)	<0.001
High salt intake	777 (63.6)	1,894 (48.2)	2,671 (51.9)	<0.001
Diet non-vegetarian	1,184 (97.0)	3,664 (93.3)	4,848 (94.1)	<0.001
Unhealthy diet habits	1,181 (96.7)	3,805 (96.9)	4,986 (96.9)	0.708
**Current tobacco use**				
All	106 (8.7)	547 (13.9)	653 (12.7)	<0.001
Men only	106 (23.7)	540 (33.5)	646 (31.3)	<0.001
**Current alcohol consumption**				
Men only	87 (19.4)	392 (24.3)	479 (23.2)	0.032
Diabetes	84 (6.9)	975 (25.0)	1,059 (20.7)	<0.001
Impaired fasting glucose	215 (17.7)	937 (24.1)	1,152 (22.5)	<0.001
Hypercholesterolemia	543 (44.9)	2,551 (65.5)	3,094 (60.6)	<0.001
**Knowledge of risk factors**				
Poor	513 (42.0)	1,753 (44.6)	2,266 (44.0)	
Others	708 (58.0)	2,176 (55.4)	2,884 (56.0)	0.113
**Checked BP at least once past year**				
Yes	287 (23.5)	1,520 (38.7)	1,807 (35.1)	<0.001
No	934 (76.5)	2,409 (61.3)	3,343 (64.9)	

### Risk Factors

Among the young adults 40.8% were obese, 58.5% had abdominal obesity, 44.9% had hypercholesterolemia, 6.9% had diabetes, 17.7% had impaired fasting glucose, 96.7% had low intake of fruits and vegetables, 23.7% of men were smokers, and 19.4% of men had consumed alcohol during the previous year. Abdominal obesity, inadequate physical activity, current tobacco use, current alcohol use (men), diabetes, impaired fasting glucose, and hypercholesterolemia were lower among the young participants compared with older participants, though there was no difference in the level of obesity. Young adults had higher prevalence of high salt intake and non-vegetarianism and there was no difference in the levels of fruits and vegetables intake (which was very low in both groups). Significantly lesser proportion of young adults had checked their blood pressure at least once in the past 1 year compared to older adults (*p*-value <0.001). However, knowledge level about risk factors for coronary artery disease was not different between younger and older participants of the study.

### Prevalence of Hypertension

Age adjusted prevalence of hypertension, prehypertension and normotension among young adults, older adults and overall study population is shown in Table 2. We found that among the young adults, one in eight had hypertension and about one third had prehypertension. Among young adults, hypertension was nearly three times more prevalent among men than women. There were no differences between men and women with hypertension with respect to urban location, below poverty line, above school education, high salt intake, and diabetes. However, physical inactivity and hypercholesterolemia were higher in men and current tobacco use and alcohol consumption were prevalent among men only. On the other hand obesity and abdominal obesity were more prevalent among women. Prehypertension was more prevalent among men in both young adults and older adults.

**Table 2:** Prevalence of hypertension, prehypertension and normotension among young adults, older adults, and overall adults by sex.

**Age group**	**Participants**	**Hypertension** ***N* (%)**	**Prehypertension** ***N* (%)**	**Normotension** ***N* (%)**
Young adults	Men (448)	92 (20.5)	213 (47.5)	143 (31.9)
	Women (773)	58 (07.5)	202 (26.1)	513 (66.4)
	*P-*value	<0.001	<0.001	<0.001
	Total (1,221)	150 (12.3)	415 (34.0)	656 (53.7)
	Age adjusted	11.2%	33.3%	55.5%
Older adults	Men (1,613)	762 (47.2)	498 (30.9)	353 (21.9)
	Women (2,316)	1,093 (47.2)	629 (27.2)	594 (25.6)
	*P-*value	1.00	0.012	0.007
	Total (3,929)	1,855 (47.2)	1,127 (28.7)	947 (24.1)
	Age adjusted	43.5%	30.2%	26.3%
Overall	Men (2,061)	854 (41.4)	711 (34.5)	496 (24.1)
	Women (3,089)	1,151 (37.2)	831 (26.9)	1,107 (35.8)
	*P-*value	0.003	<0.001	<0.001
	Total (5,150)	2,005 (37.2)	1,542 (29.9)	1,603 (31.0)
	Age adjusted	27.9%	31.7%	40.4%

Table 3 shows the factors influencing hypertension in the young adults in bivariate and age adjusted multivariate analysis. Male sex, urban residence, abdominal obesity and hypercholesterolemia increased the risk of hypertension in young adults in the study.

**Table 3:** Prevalence of hypertension by socio-economic status and clinical risk factors in young adults: results of bivariate and age adjusted multivariate analysis.

**Characteristics**	**Prevalence of hypertension** ***n* (%)**	**Adjusted OR** **(95% CI)**	***P-*value**
**Sex**			
Women	58 (7.5)	Reference	<0.001
Men	92 (20.5)	3.36 (2.15–−5.25)	
**Place of residence**			
Rural	64 (8.3)	Reference	<0.001
Urban	86 (18.9)	2.21 (1.52–3.22)	
**Poverty level**			
Above poverty line	80 (11.1)	Reference	0.064
Below poverty line	70 (13.9)	1.45 (0.97–2.15)	
**Educational status**			
Above school education	56 (10.9)	Reference	0.290
Up to school education	94 (13.3)	1.25 (0.82–1.92)	
**Salt intake**			
High	86 (11.1)	Reference	0.017
Not high	64 (14.4)	1.60 (1.08–2.37)	
**Current tobacco use**			
No	124 (11.1)	Reference	0.519
Yes	26 (24.5)	1.65 (0.36–7.55)	
**Current alcohol consumption**			
No	97 (9.6)	Reference	0.353
Yes	53 (25.1)	2.12 (0.43–0.37)	
**Physical activity**			
Non-sedentary	117 (11.3)	Reference	0.444
Sedentary	33 (18.0)	1.12 (0.74–1.97)	
**Obesity**			
No	71 (9.8)	Reference	0.174
Yes	79 (15.9)	1.37 (0.87–2.15)	
**Abdominal obesity**			
No	47 (9.3)	Reference	0.028
Yes	103 (14.5)	1.74 (1.06–2.87)	
**Diabetes**			
No	129 (11.4)	Reference	0.204
Yes	20 (23.8)	1.47 (0.81–2.66)	
**Hypercholesterolemia**			
No	55 (8.3)	Reference	0.011
Yes	90 (16.6)	1.64 (1.12–2.40)	

### Awareness, Treatment, and Control

**Figure 1:**
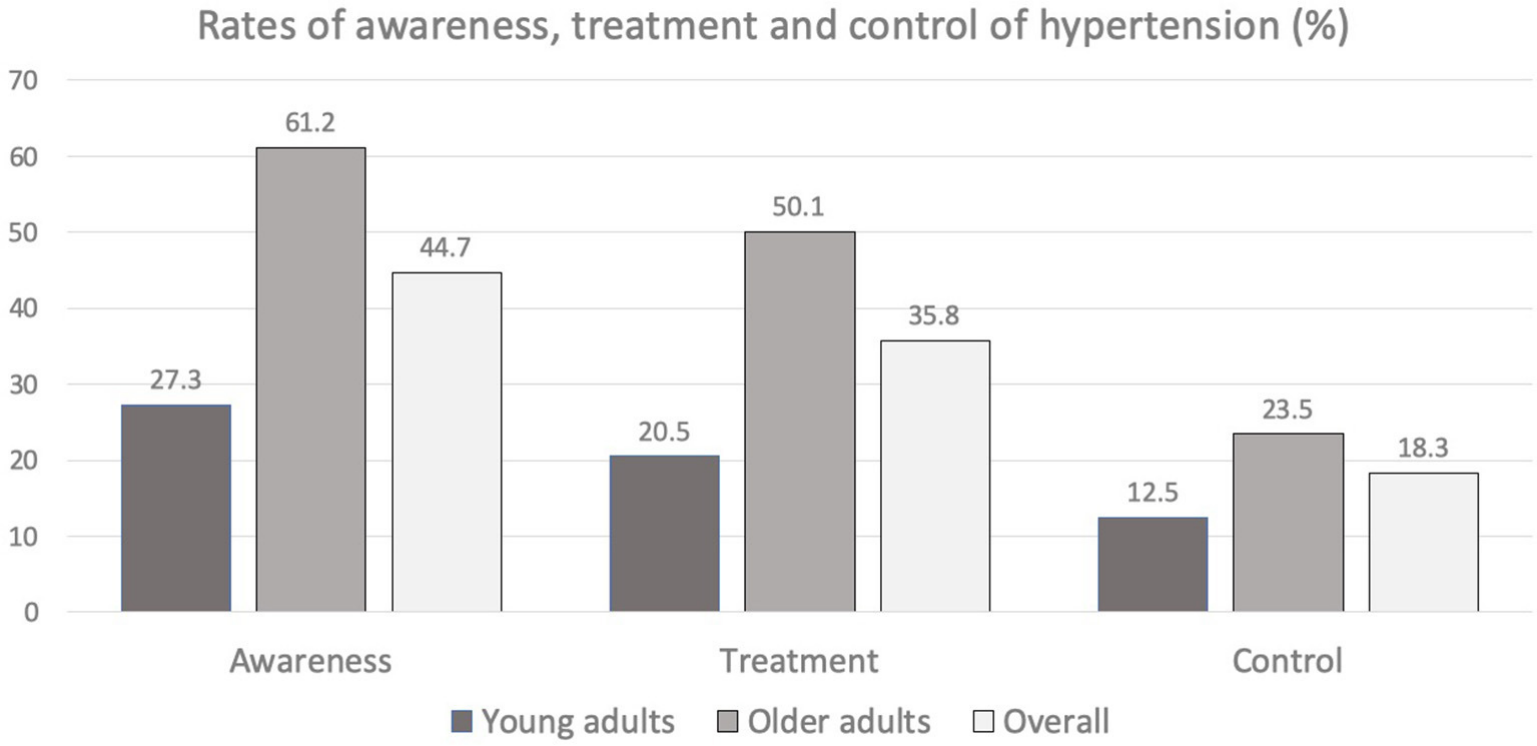
Rates of awareness, treatment and control of hypertension among young adults and older adults.

We looked at the awareness, treatment and control of hypertension in the three groups (Figure 1; Table 4). Awareness and treatment of hypertension were significantly poor among young adults compared to older adults (awareness 34.7 vs. 64.0 *p*-value <0.001 and treatment 26.0 vs. 54.0 *p*-value <0.001, respectively). There was also a trend toward reduced control of hypertension among younger adults compared to older adults (17.3 vs. 23.8 *p*-value 0.072). Awareness, treatment and control of hypertension were significantly lower among men compared to women (23.9 vs. 51.7 with *p*-value 0.001, 12.0 vs. 25.9 with *p*-value 0.045, and 18.5 vs. 37.9 with *p*-value 0.012, respectively). These parameters were also significantly less among men compared to women in the older age group as well as overall hypertensive population.

**Table 4:** Awareness, treatment and control of hypertension among younger adults, older adults, and overall hypertensive population.

**Age group**	**Hypertension**	**Awareness** ***N* (%)**	**Treatment** ***N* (%)**	**Control** ***N* (%)**
Young adults	Men (92)	22 (23.9)	17 (18.5)	11 (12.0)
	Women (58)	30 (51.7)	22 (37.9)	15 (25.9)
	*P-*value	0.001	0.045	0.012
	Total (150)	52 (34.7)	39 (26.0)	26 (17.3)
	Age adjusted (total)	27.3%	20.5%	12.8%
Older adults	Men (762)	451 (59.2)	365 (47.9)	158 (20.7)
	Women (1,093)	738 (67.5)	637 (58.3)	284 (26.0)
	*P-*value	<0.001	0.009	<0.001
	Total (1,855)	1,189 (64.1)	1,002 (54.0)	442 (23.8)
	Age adjusted (total)	61.2%	50.1%	23.5%
Overall	Men (854)	473 (55.4)	382 (44.7)	169 (19.8)
	Women (1,151)	768 (66.7)	659 (57.3)	299 (26.0)
	*P*-value	<0.001	<0.001	0.001
	Total (2,005)	1,241 (61.9)	1,542 (51.9)	1,603 (23.3)
	Age adjusted (total)	44.7%	35.8%	18.3%

*P-values for awareness, treatment and control between young and older adults: <0.001, <0.001, and 0.072, respectively*.

We also looked at other possible factors contributing to the low awareness, treatment and control of hypertension among the young adults (Table 5). Our results showed that treatment and control of hypertension were better if they were obese and more physically active. Those who had checked their blood pressure at least once past year had significantly better awareness and treatment with a trend toward better control of hypertension. Participants from rural areas had better control of hypertension than those from urban areas, though levels of awareness and treatment were not different. Surprisingly, abdominal obesity, education status, knowledge level of risk factors for CAD and poverty level did not influence these parameters.

**Table 5:** Awareness, treatment, and control of hypertension among the young adults in relation to baseline characteristics.

**Parameter**	**Hypertension**	**Awareness** ***N* (%)**	**Treatment** ***N* (%)**	**Control** ***N* (%)**
Sex	Men (92)	22 (23.9)	17(18.5)	11 (12.0)
	Women (58)	30 (51.7)	22 (37.9)	15 (25.9)
	*P-*value	0.001	0.045	0.012
Place of residence	Urban (86)	28 (32.6)	20 (23.3)	10 (11.6)
	Rural (64)	24 (37.5)	19 (29.7)	16 (25.0)
	*P-*value	0.604	0.452	0.045
Education	Up to school education (94)	38 (40.4)	29 (30.9)	20 (21.0)
	Above school education (56)	14 (25.0)	10 (17.9)	6 (10.7)
	*P-*value	0.076	0.087	0.121
Poverty level	Above poverty line (80)	29 (36.3)	20 (25.0)	13 (16.3)
	Below poverty line (70)	23 (32.9)	19 (27.1)	13 (18.6)
	*P-*value	0.732	0.853	0.121
Knowledge level of risk factors	Poor (54)	14 (25.9)	11 (20.4)	7 (13.0)
	Others (96)	38 (39.6)	28 (29.2)	19 (19.8)
	*P-*value	0.109	0.253	0.371
Checked BP at least once past year	Yes (46)	27 (58.7)	19 (41.3)	12 (26.1)
	No (104)	25 (24.0)	20 (19.2)	14 (13.5)
	*P-*value	<0.001	0.008	0.066
Physical activity	Sedentary (33)	7 (21.2)	4 (12.1)	3 (9.1)
	Non sedentary (117)	45 (38.5)	35 (29.9P)	23 (19.7)
	*P-*value	0.096	0.044	0.020
Obesity BMI ≥ 25	Yes (79)	33 (41.8)	28 (35.4)	20 (25.3)
	No (71)	19 (26.8)	11 (15.5)	6 (8.5)
	*P-*value	0.060	0.009	0.009
Abdominal obesity	Yes (47)	40 (38.0)	31 (30.1)	19 (18.4)
	No (103)	12 (25.0)	8 (17.0)	7 (14.9)
	*P-*value	0.140	0.110	0.650

## Discussion

### Prevalence of Hypertension

In this community survey of CAD and risk factors for CAD conducted in three rural and three urban areas selected from north, central and southern regions of Kerala, we found that age adjusted prevalence of hypertension was 27.9% which was comparable to overall prevalence of hypertension of 30% noted in a large meta-analysis which included major studies from India (2) and the prevalence of 29% reported in the US National Health and Nutrition Examination Survey (NHANES) 2015-16 (15). A recent community based cross sectional survey from all districts of Kerala also showed a prevalence of raised blood pressure of 30.4% (16).

### Prevalence of Hypertension in the Young

Age adjusted prevalence of hypertension among young adults was 11.2% in our study which was higher than that reported from the developed world. NHANES 2015-16 reported age adjusted prevalence of hypertension of 7.5% among young adults in the age group 20–39 years in US (15). In a comparative study of prevalence of hypertension in England, USA and Canada, Joffres et al. reported prevalence of hypertension among the young adults of 9.3% in England, 7.7% in USA and 2.0% in Canada while overall prevalence of hypertension was 30.0, 29.1, and 19.5%, respectively (17). Many studies from India have reported prevalence of hypertension among the young adults varying from 11 to 22.7% (18–24). Wide variations in the prevalence of hypertension in young subjects in these studies are mostly due to differences in the population screened, age group included, definition employed and lack of age standardization. In general, however, prevalence of hypertension among young adults in India is much higher than those reported from the developed economies. This higher prevalence is in consonance with the increased prevalence of risk factors for CAD at younger age in India (25, 26). This was also evident from the prevalence of various risk factors for CAD in the present study mentioned before in the results area.

Based on our estimate of age adjusted prevalence of hypertension of 11.2% among young adults, India with a population of 387 million in the age group 20–39 years as per census 2011 (men 197 million and women 190 million) will have 43 million young individuals with high blood pressure, a huge burden for a resource poor country (3). In addition, one third of the young adults had prehypertension, a condition which can progress to hypertension and this emphasizes the need to promote lifestyle changes among the young adults to prevent the future development of frank hypertension. Prehypertension was more prevalent among them compared to older adults. Similar findings were observed by Gupta et al. (24). They found prevalence of prehypertension in 45.1% of men and 30.4% of women among the young adults, compared to 33.8 and 28.6%, respectively, among older adults. Predictors of hypertension in the young adults in our study apart from male sex were urban residence, abdominal obesity and hypercholesterolemia which points to lifestyle factors leading to the development of hypertension in the young.

### Prevalence in Men vs. Women

Another striking finding in our study was the three times higher prevalence rate of hypertension among young men compared to young women. Interestingly this male preponderance was not seen in the older adults and the male preponderance in the overall hypertensive population was mainly due to the higher prevalence in the younger men. In the US data from NHANES for 2015-16, there was higher prevalence of hypertension in men compared to women in the age group 18–39 years, but the difference was much less (15). The prevalence of hypertension in that study was 9.2% in men and 5.6% in women in the age group 18–39 years, while it was 30.2% in men and 27.7% in women in the overall study population. Similarly in the study by Gupta et al. (24) there was significant difference in the prevalence of hypertension in men and women in the young subjects, but not in the older subjects (22.3 and 18.7% in men and women, respectively, in the 20–39 years age group vs. 50.7 and 51.0% in men and women, respectively, in the older subjects). The difference between the men and women in the young adults in the above study was less striking than the difference found in our study. Reasons why young men have such a high prevalence of hypertension compared to women is to be further explored. Possible factors contributing to this are higher levels of alcohol consumption and smoking, hypercholesterolemia and less physical activity and unlikely to be due to differences in location of residence, education, poverty level, obesity or abdominal obesity. The older men in our study had higher prevalence of smoking and alcohol consumption than younger men, yet they didn't show higher prevalence of hypertension compared to women. It has been suggested that the differences in the prevalence of hypertension between young men and women may be explained by the effects of oestrogens and androgens on BP modulation (27). In any case, public health efforts should target the young men in promoting adoption of healthy lifestyle to prevent development of hypertension.

### Awareness, Treatment, and Control

The levels of awareness, treatment and control of hypertension in young adults as well as older adults were substantially lower compared to that reported from the high income countries (28). The lower levels of awareness, treatment and control of hypertension were more evident in the young hypertensive population. In NHANES 2013-14, awareness was 74.7% (27.3% in our study), treatment was 50.0% (20.5% in our study) and control was 40.2% (12.8% in our study) in the young adults in the 20–39 years age group (28).

### Awareness, Treatment, and Control: Men vs. Women

The awareness, treatment and control of hypertension were worse in men compared to women and similar trend was noted in the US study as well. In our study, control rate of hypertension in young men was 12.0% compared to 25.9% in young women and this was also evident in the NHANES 2013-14 survey as well which showed control rate of 33.7 and 51.8% in men and women, respectively (age group 18–39 years). The higher awareness, treatment and control rates of hypertension in women is most likely due to more health care visits by young women though frequency of health care visits was not captured in our study. An analysis of data showed that more frequent healthcare visits in young women explained 52% of the difference in control of hypertension between women and men in NHANES 2013-2014 (29). Better awareness and treatment in those young subjects who had checked their blood pressure at least once during the previous year shows that if facilities to check blood pressure can be improved and if the public is educated regarding the necessity of checking blood pressure periodically, control of blood pressure is likely to improve. The better treatment and control of hypertension in those with obesity could be due to more opportunities for coming into contact with the health system or fear of developing hypertension or its complications.

## Strengths and Limitations

Our study is the first population survey from India that reports prevalence of hypertension among young adults. The study shows that high blood pressure is highly prevalent among young adults in India. The gender differences in its prevalence, awareness, treatment and control found in our study have not been highlighted in any previous Indian studies. Main limitation of the study is that it is a regional study done in a small state in India and the findings may not be applicable to rest of India. Another limitation is that blood pressure is measured at one time point only and this may overestimate the true prevalence of hypertension in this population.

## Conclusions

Our study showed that one out of nine young adults and more than a quarter of overall adult population have high blood pressure. Additionally, prehypertension was found in one third of the young adults, which was higher than in the older adults. We found nearly three times higher prevalence of hypertension in young men compared to young women, possibly related to lifestyle factors and protective effects of sex hormones in the premenopausal women. The levels of awareness, treatment and control of hypertension in young adults were substantially lower compared to the levels in the older adults. The control of hypertension was poor in young men compared to women. Awareness, treatment and control of blood pressure was better when young subjects had checked their blood pressure at least once during the previous year and when knowledge level of risk factors was high. Identifying young adults with hypertension and prehypertension is important since hypertension is a modifiable risk factor for development of CAD. Young men have higher risk of hypertension and prehypertension and should receive proper health education and support. Proper management of hypertension in young adults is especially relevant in India, a country with a sizable young population.

## Data Availability Statement

The original contributions presented in the study are included in the article/supplementary material, further inquiries can be directed to the corresponding author.

## Ethics Statement

The studies involving human participants were reviewed and approved by Cardiological Society of India Independent Ethics Committee. The patients/participants provided their written informed consent to participate in this study.

## Author Contributions

ZG conceived and designed the study, contributed to data collection and analysis, and writing of the paper. GM did the statistical analysis and contributed to writing of the manuscript. KV, PM, SH, and GS contributed to the collection of the data and its analysis and writing of the paper. KT conceived and designed the study and contributed to the analysis and writing of the paper. All authors contributed to the article and approved the submitted version.

## Funding

Funded by unrestricted Grant from Cardiological Society of India, Kerala Chapter. Open access publication fees are also paid from the funds from Cardiological Society of India Kerala Chapter.

## Conflict of Interest

The authors declare that the research was conducted in the absence of any commercial or financial relationships that could be construed as a potential conflict of interest.

## Publisher's Note

All claims expressed in this article are solely those of the authors and do not necessarily represent those of their affiliated organizations, or those of the publisher, the editors and the reviewers. Any product that may be evaluated in this article, or claim that may be made by its manufacturer, is not guaranteed or endorsed by the publisher.

## Appendix

**Knowledge Level of Risk Factors Questions:**

1. Do you think smoking causes

- Heart Attacks
- Stroke
- Cancers
- Don't know

2. Do you think high blood pressure causes

- Heart Attacks
- Stroke
- Cancers
- Don't know

3. Do you think one has high blood pressure if

- Systolic BP > 140 and/or diastolic BP > 90
- Systolic BP > 160 and/or diastolic BP > 95
- Systolic BP > 180 and/or diastolic BP > 100
- Don't know

4. Do you think cut off for normal BP in an adult depends on age?

- Yes
- No

5. What is the most important dietary intervention to control high blood pressure?

- Avoid coconut oil
- Eat vegetarian diet
- Eat fruits and vegetables
- Restrict salt
- Don't know

6. Do you think diabetes causes

- Heart attacks
- Kidney disease
- Strokes
- Cancers
- Don't know

7. Desirable level of total cholesterol in a person without heart disease is

- <200 mg/dL
- <230 mg/dL
- <250 mg/dL
- Don't know

Answer Key 1: ABC 2: AB 3: A 4: B 5: D 6: ABC 7: A Score one point for each correct answer If score 5 to 7: Good knowledge level of risk factors If score 2 to 4: Satisfactory knowledge level of risk factors If score 0 to 1: Poor knowledge level of risk factors
